# Supplementary material for: Nanoscale Mapping of Magnetic Auto-Oscillations with a Single Spin Sensor
Source: Nano Lett. 2025 Jan 22;25(5):1917–24. doi: 10.1021/acs.nanolett.4c05531 (PMC11803721; doi:10.1021/acs.nanolett.4c05531)
Supplement: Supplementary file 1 — nl4c05531_si_001.pdf [file nl4c05531_si_001.pdf]

Supplementary Information for
Nanoscale Mapping of Magnetic Auto-Oscillations with a Single Spin Sensor

Toni Hache[1,2*], Anshu Anshu[1], Tetyana Shalomayeva[2], Gunther Richter[3], Rainer Stöhr[2], Klaus Kern[1,4], Jörg Wrachtrup[1,2,5], Aparajita Singha[1,5,6*]

[1*]Max Planck Institute for Solid State Research, Heisenbergstr. 1, Stuttgart, 70569, Germany.
[2]3rd Institute of Physics and Research Center SCoPE, University of Stuttgart, Stuttgart, 70049, Germany.
[3]Max Planck Institute for Intelligent Systems, Heisenbergstr. 3, Stuttgart, 70569, Germany.
[4]Institute de Physique, École Polytechnique Fédérale de Lausanne, Lausanne, CH-1015, Switzerland.
[5]Center for Integrated Quantum Science and Technology IQST, University of Stuttgart, Stuttgart, 70049, Germany.
[6]Technical University of Dresden, Institute of Solid State and Materials Physics & Wurzburg Dresden Cluster of Excellence, Dresden, 01062, Germany.

*Corresponding author(s). E-mail(s): t.hache@fkf.mpg.de; a.singha@fkf.mpg.de, aparajita.singha@tu-dresden.de;

1 Electrical measurements of Auto-oscillations

In order to extract the auto-oscillation signals from the all-electrical measurements (Fig. 1(f) of the main manuscript) a background subtraction algorithm was used. After acquiring the data by the spectrum analyzer as function of the dc current, each frequency channel was fitted to a quadratic function with subsequent subtraction (Fig. SI 1(a)). This removes signals stemming from standing waves within the microwave circuit and the noise resulting from Joule heating. Figure SI 1(b) shows that auto-oscillation are excitable only at positive dc currents due to the symmetry of the spin Hall effect. A reversal of the dc current polarity results in the switching of the spin current polarity. This flips the the direction of the spin-orbit torque which results to an increased damping in the system and, therefore, no auto-oscillations can be generated. Figure SI 1(c) shows a single spectrum of SI 1(b) at 8.5 mA.

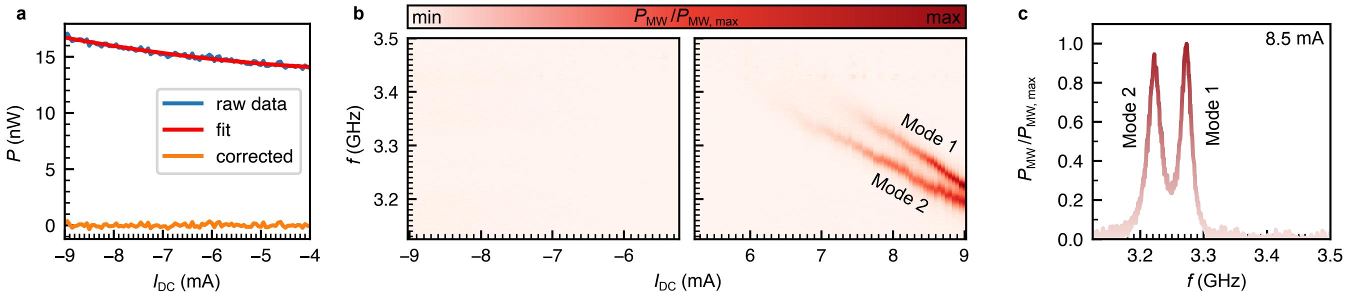

Figure SI 1: a) Example of background subtraction from the all-electrical measurement. The increase of the background noise level as a function of dc current is a result of the increasing Joule heating. b) Generation of auto-oscillations is achieved only for positive dc currents. At negative currents, the sign of the SOT is reversed and the damping in the system is increased prohibiting the formation of auto-oscillations. c) Single spectrum at 8.5 mA.

2 Height dependence of the magnetic stray field component parallel to the NV axis

We estimated the distance of the NV sensor to the SHNO by comparing shape and values of the calculated magnetic stray field to the measured distribution shown in Fig. 2 of the main manuscript. Figure SI 2 shows the calculated magnetic stray field generated by the $Ni_{81}F_{19}$ inside the SHNO at different heights. The contrast between magnetic field minima within the constriction and the surrounding area becomes smaller with increasing distance from the sample. The shape and the values of the magnetic field at 80 nm fits best to the quantified stray field in the experiment.

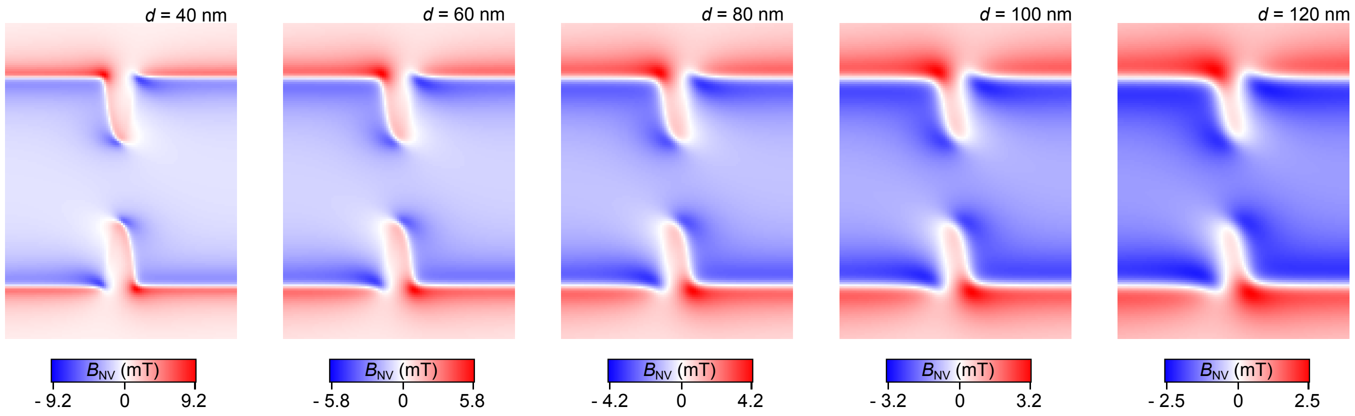

Figure SI 2: Calculated magnetic stray field along the NV axis at different distances above the SHNO.

3 PL maps before background subtraction

The measurement data shown in Fig. 4 of the main manuscript was obtained after two evaluation steps. 1) Normalization of the photon count rate of each row by an averaged value outside of the constriction area in order to exhibit the PL contrast on the maps. 2) Subtraction of the PL map without applied dc current to remove any spurious other effect influencing the photon count rate apart from the auto-oscillations. Figure SI 3(a) shows the mapped photon count rate at each pixel at $I_{DC} = 0$ mA. There is a slight reduction of the photon count rate at the top left area which isn't caused by magnetic auto-oscillations and, therefore, this map is subtracted from the maps with auto-oscillations. Figure SI 3(b) and (c) show the PL maps (before background subtraction) when the auto-oscillations are active.

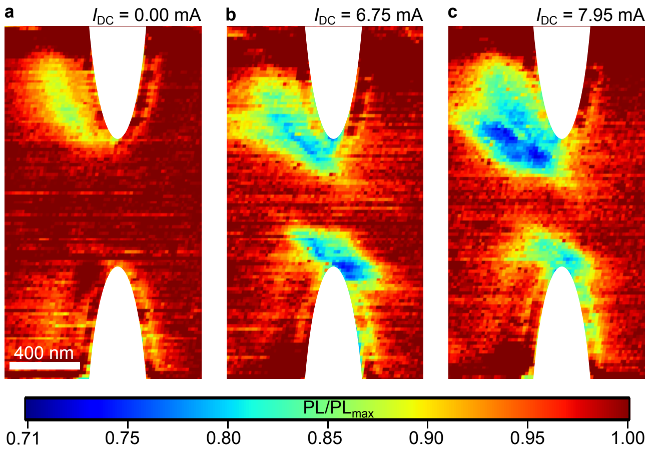

Figure SI 3: Measured PL maps before background substraction. (a) Background map at $I_{DC} = 0$ mA with a slightly reduced photon count rate at the top left area. (b), (c) PL maps (before background subtraction) when the auto-oscillations are active.

4 Microwave field components generated by a single precessing spin

In order to get a deeper understanding of the microwave field generation caused by the auto-oscillations (Fig. 4 f) to i)) we calculated the microwave generation 80 nm above a single precessing spin. For each time step, the dipolar magnetic field was calculated resulting in time-dependent dipolar field data. The FFT of this time-dependent field gives the dynamic components at the precession frequency. To emulate the elliptical precession of the magnetic moment in a

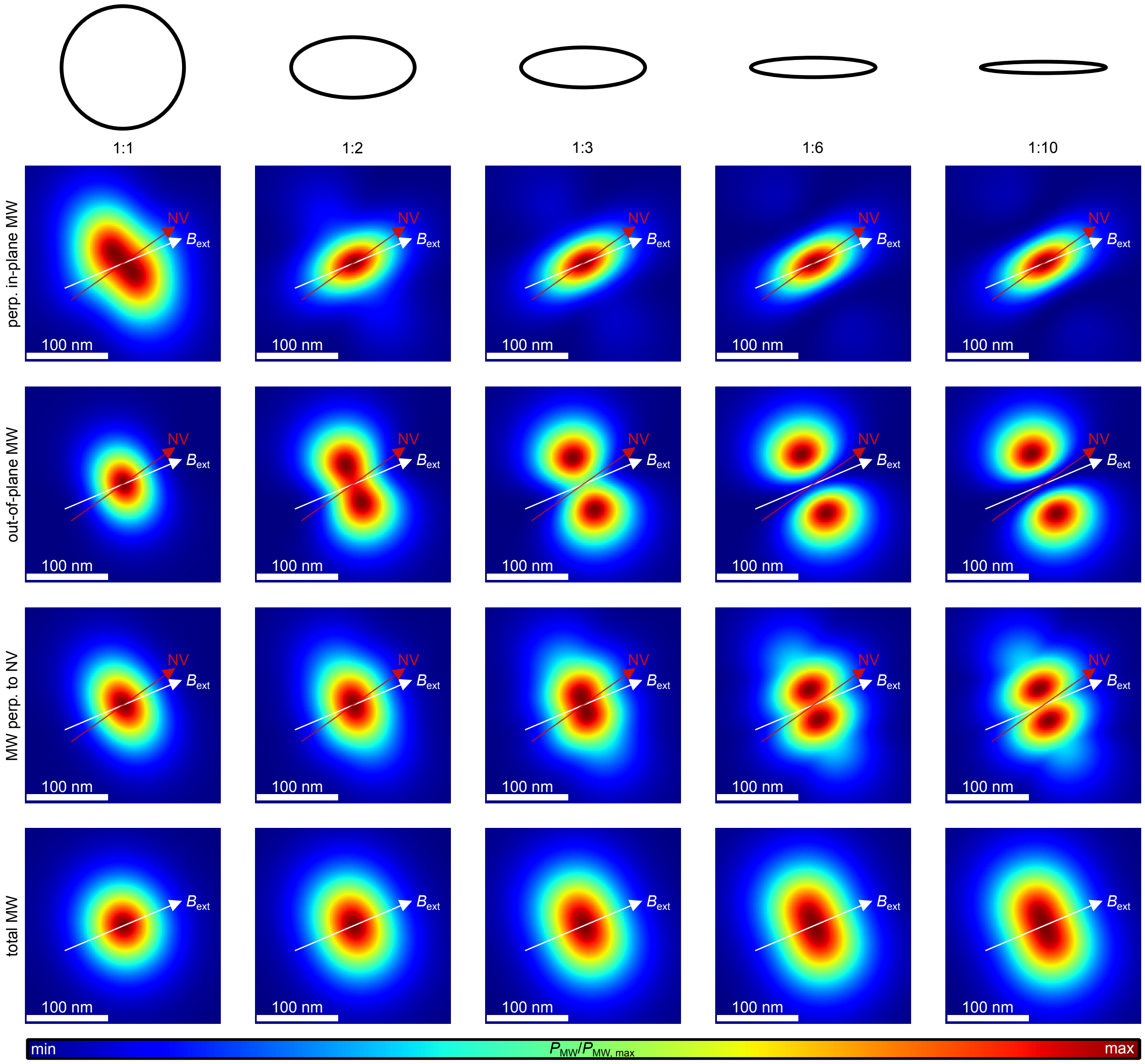

Figure SI 4: Calculated microwave field components of a single precessing magnetic moment calculated at a distance of 80 nm. Arrows indicate the alignment of the magnetic field and the NV axis which correspond to the angles in the experiment.

thin film sample caused by the shape anisotropy, different ratios of the minor and major axis of the ellipse from 1:1 to 1:10 are used (first row of Fig. SI 4). With increasing ellipticity of the precession the out-of-plane component of the microwave field shows an increasing separation of two maxima. The in-plane component shows always only one maximum independent of the ellipticity of the precession cone. The total microwave field which can interact with the NV shows similar to the out-of-plane component two maxima with increasing separation for stronger ellipticity. The total microwave field power has always only one maximum. The shape of the total microwave power distribution becomes more anisotropic for increased ellipticity. The elliptical precession with a ratio 1:6 correspond to the precession behavior of the auto-oscillating magnetic moments shown in Fig. 4(e) in the main manuscript. Based on this calculations

we conclude that the formation of two maxima in Fig. 4 g) and h) of the main manuscript is caused by the elliptical precession of the magnetic moments.

5 Control of auto-oscillation areas via external magnetic field angle

Micromagnetic simulations are conducted to investigate the dependence of the auto-oscillation formation on the position of the internal magnetic field minima (spin-wave potential wells) in $Ni_{81}F_{19}$. Figure SI 5(a) shows how the magnetic field minima shift along the edge of the constriction to the right when the the external magnetic field angle is increased from 0° to 60°. Figure SI 5(b) shows the position of the auto-oscillations at the constriction edge at these field angles, respectively. It can be clearly seen, that the auto-oscillation spots follow the internal magnetic field minima. As mentioned in the main manuscript, the asymmetry of the measured auto-oscillation spots with respect to the sample geometry is caused by the shift of the internal magnetic field minima defined by the external magnetic field angle.

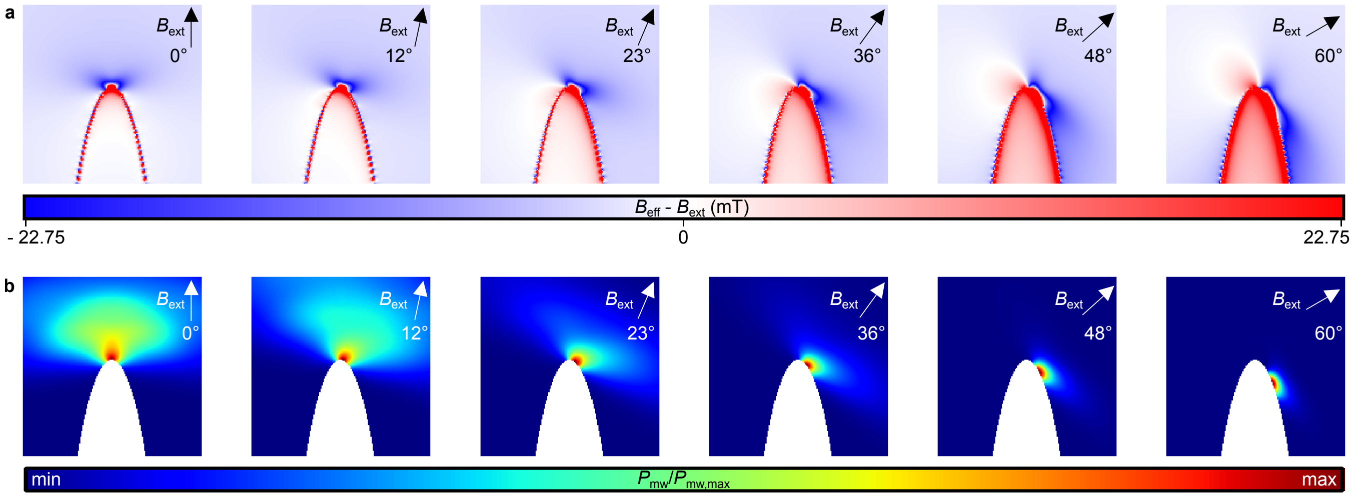

Figure SI 5: The in-plane angle of the external magnetic field defines the position of the internal magnetic field minima as shown in (a). The auto-oscillation areas shown in (b) follow the position of these internal magnetic field minima which act as potential wells for spin-waves.

6 Shape of the SHNO for the micromagnetic simulation

A micromagnetic simulation is conducted in order to analyze the microwave field generated above. A small asymmetry is introduced in order to emulate the behavior in the experiment for which both auto-oscillation modes at the edges show different frequencies and, therefore aren't mutually synchronized. The SHNO shape is shown in Fig. SI 6. The asymmetry leads to slightly different auto-oscillation frequencies of 3.62 GHz at the bottom constriction edge and 3.65 GHz at the top constriction edge. The auto-oscillation mode profile at the bottom constriction is shown in Fig. SI 6. This mode is shown in Fig. 4(e) of the main manuscript and was used as input for the microwave field calculations shown in Fig. 4(f) to (i). This simulation reveals that already small deviations from the mirror symmetry of the constriction has a strong influence on the overall spectral characteristic of the device. In the experiment, this might be amplified by variations in the saturation magnetization, for instance, due to different oxidation at the edges.

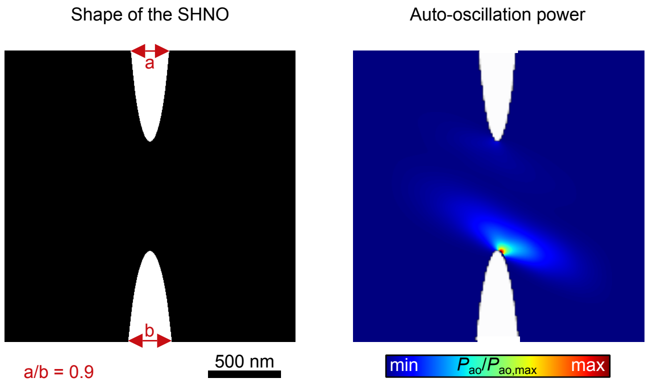

Figure SI 6: The asymmetric shape leads to distinct auto-oscillation frequencies at both constriction edges. The auto-oscillations at 3.62 GHz are located at the bottom constriction edge with the shown mode profile.
